# Sketches of the Introductory Lectures of Bell, Conolly, Davis, Pattison, Thomson, Watson, and Turner; with General Remarks on the Objects, Tendency, and Probable Consequences of This Institution

**Published:** 1829-01-01

**Authors:** 


### LONDON UNIVERSITY.

ON Wednesday, the first of October, the LONDON UNIVERSITY opened. MEDICINE had the honour of leading the Van—and Mr. Charles Bell had the honour of representing MEDICINE. The spacious theatre dedicated to this department of knowledge was crowded to excess, long before Mr. Bell appeared—and great numbers were unable to obtain admittance. There could not be less, we imagine, than 800 persons present. We were sorry to observe so few of the PRIMATES of the profession there. We should suppose that about a third of the audience—perhaps more, were non-medical. The great mass of the audience, as far as we could observe, consisted of the middle ranks of the profession—the upper classes of general practitioners, in whom, perhaps, will be found, after all, the greatest share of good sense, liberal feeling, public spirit, and professional information. There was a sprinkling, but a very sparse one, of those who claim the higher walks of physic and surgery. There were at least five physicians to one surgeon (we mean the *Pures* of both descriptions) present, although the professor was a surgeon, and consequently the subject surgical. The front seat was occupied by civilians, among whom he noticed Sir James Macintosh—the second tier was principally filled with professors in their robes—all above these, up to the summit of that vast theatre, consisted of a *melé* of all ranks of medical society, as well as non-professional visitors.

When Mr. Bell entered, the most astounding applause instantly arose from every part of the theatre. This gratifying perturbation over, Mr. Bell proceeded to deliver, principally from a written paper, a neat and appropriate address. He declared, that the circumstance of *his* appearance—as the FIRST to open the lectures of the new institution, was *purely accidental*—an assertion probably more founded in modesty than truth. In stating what he hoped and believed would be the advantages of such an institution as the London University, (as far as the medical department was concerned,) he did not disguise the difficulties by which it was surrounded. The numerous rival schools of medicine and surgery with which this great metropolis is studded, rendered it clear that the success of the institution depended on the strenuous exertions of the professors themselves. They were placed on an acclivity—or rather, in a rapid stream. If they did not ascend, they must inevitably be swept down into the gulph of oblivion!

> "Like one who, 'gainst a stream's impetuous course,
> Impels his slow boat, urged with all his force,
> If once his vigour fail, or arms grow slack,
> With headlong force the torrent whirls him back." *Virg.*

Mr. Bell contrasted the schools of medicine in London with those of the regular Universities. In the *latter*, the professors were stipendiaries, and consequently rendered independent of private practice. In such schools, THEORY would naturally prevail. In London, where the teachers had to depend on their own industry and talents, tuition was generally made a step to the more lucrative avocations of private practice; and when the latter was attained, the former was, of course, abandoned. The consequence of such a state of things was, that the metropolitan teachers of medicine and surgery were generally a succession of young men who, in proportion as they increased in experience, moved off from the ranks of lecturers to swell the army of practitioners. In such schools, *practical facts* would, of course, be more in requisition than abstract reasoning or theory. The medical constitution of the LONDON UNIVERSITY would, it was hoped, unite the advantages of both systems, and leave their defects behind. It was to be hoped that such an institution would secure men of talent—of established reputation—of practical experience—of moral worth, as its PROFESSORS—while it would be able to hold out temptations to the most active exertions of genius and industry among its pupils.*

* We suppose this alluded to the plan

Here Mr. Bell drew a picture of the cheerless desultory life of the London medical student, attendant on the private schools. Completely his own master, and without supervisorship or religious instruction, he is left to the guidance of his own unassisted reason—and what is worse, to the impulse of uncontrolled passions, too generally with a very imperfect preliminary education. What the consequences of such a situation may be, it is not very difficult to imagine. The London University provides for moral and religious instruction, and its professors will see that such instruction is attended to, by frequent examination of the pupils under their charge.

But the grand advantage of the institution is the facility which it affords of combining classical and scientific literature and knowledge with that which is purely medical. This is an advantage on which we have often dwelt, and on which we need not now descant. The utility of such institutions as the one under consideration, gave Mr. Bell an opportunity of throwing in a passage which called forth warm plaudits from the audience.

" At present, while this beautiful edifice is incomplete, and the labours of the workmen are only suspended, and clamour is excited, it requires some exertion of the mind to rid itself of the influence of these circumstances, and fully to anticipate and appreciate the advantages to be derived from this College—*and not from this College only, but from others formed after its example, by the exertions of those who, although they may not have had the genius to conceive the plan, yet may have had the virtue to imitate it.*" GREAT APPLAUSE.

We apprehend that most people who look beyond the surface of things, will readily see that it was not so much the *want of genius* to plan a similar institution, as the *disinclination* to give it existence, which prevented the projection of a KING'S College—till a PEOPLE'S College was on the point of being roofed in.

We shall not, in this place, take into consideration the physiological illustrations which were introduced in his discourse. The application of hydraulics to the circulation of the blood was neatly and ingeniously demonstrated. We by no means agree with Mr. Bell in his ideas respecting the *alternate* dilatation and contraction of the trunks of arteries, as aids to the circulation of the blood; and we have often dissented from him on this point of physiology; but this is not the place to renew the discussion. His introductory lecture was so well received, that his successor, on the second day, had a somewhat fearful task to encounter. The result we shall now, with pleasure, state.

Dr. CONOLLY opened the second sitting with an introductory lecture to his course on the nature and treatment of diseases. The audience was nearly as great as on the first opening of the University, under the auspices of Mr. Bell—and, strange to say, there were considerably more of the *magnates* of the profession present. Whether this was occasioned by the public and private reports of Mr. Bell's preliminary discourse—or by the curiosity excited by "a first appearance on any stage," we shall not attempt to determine; but such was the fact.

It is hardly necessary to pass a greater eulogy on Dr. Conolly's introductory lecture, than to state that it drew forth the most thundering plaudits from the audience, and that it has been printed by order of the Council. It gives us exceeding pleasure to find that the Council, in appointing professors whose names had not previously been enrolled on the "lists of fame," have evidently acted on good information (however derived) respecting the talents and acquirements of those whom they entrusted with chairs in the new institution. In allusion to the concluding passage in Dr. Conolly's address, we cannot help congratulating the UNIVERSITY on possessing a man whose virtue spurns the praise of the radical press, and whose courage defies its defamatory abuse. The passage in question excited unmixed applause from every bench in the theatre. This circumstance is sufficiently indicative of the altered temper of the times. Happily for the credit of the profession and the good of society, the tide of evil passions is ebbing with a velocity and impetus which no human

---

of selecting demonstators, &c. from among the most distinguished pupils.—*Ed.*

power, however diabolical, can stem or retard! As well might they attempt to arrest the course of the majestic Rhine in its calm descent into the Ocean, and hurl it back to its ten thousand sources leaping from crag to crag among the Alps!

On the third day, Dr. Davis acquitted himself admirably in his introductory lecture to the important department of midwifery, and called down repeated plaudits from every part of a crowded theatre.

Mr. Granville Sharpe Pattison presented himself on the fourth day, as Professor of Anatomy. Although a British subject, he may be said to be a stranger in England, and, on this account, as well as on account of some recent transactions, had difficulties—perhaps prejudices to encounter, which could not fail to call forth the sympathy of every liberal mind. Happily, Mr. Pattison required no aid from sympathy. As far as could be judged by an introductory lecture, this gentleman stood *inferior to none* who had preceded him, whether we consider the matter or the manner of the lecture. His discourse was learned, yet animated—and his manner proved that he was quite at home in the situation of a public lecturer. His reception must have been exceedingly gratifying to himself and friends.

Dr. A. T. Thomson was the fifth on the list. This gentleman's well-known talents—his general attainments in medical science and its collateral branches—but especially his great proficiency in botany, chemistry, pharmacy, and materia medica, had created an anticipation in the public mind which was amply realized. Even in the narrow limits of a preliminary discourse, Dr. Thomson displayed such an accurate acquaintance with the history, the literature, and the science of his department, as drew down unequivocal testimony of approbation from every corner of the theatre, in the shape of long and loud acclamations!

Last of all, appeared, before a most crowded audience, Dr. Watson, Professor of Clinical Medicine, and one of the physicians to the Middlesex Hospital. His youth, his modesty, his good sense, the eloquence of his discourse, and, above all, the excellent matter which it contained, commanded universal approbation —nay, admiration! No man could have more forcibly portrayed the utility—the indispensible necessity of clinical instruction, than did Dr. Watson. Dr. W. manfully yet feelingly touched upon the unaccountable defect in those public bodies who have the regulation of medical education committed to their care, as far as regards clinical instruction—the most important portion of all the studies of the physician and surgeon. True it is, that the College of Surgeons and the Company of Apothecaries enjoin a certain number of *rounds or runs* in some hospital or infirmary, which are more calculated to burst a blood-vessel in the pupil's chest, than lay in a store of practical information in his head. They may say, indeed, how can we enforce clinical *lectures* when there are so few clinical *lecturers?* To this we reply, not precisely in the words, but in the spirit of Dr. Watson's discourse—make *clinical instruction* an item in the curriculum, and clinical *lecturers* will leap into existence, in every hospital of this great metropolis.

We have now to make a few observations on the opening of this national theatre of instruction. In our last number we dedicated a short article to this subject—and while the friends of the LONDON UNIVERSITY were despairing—its enemies despising—and the public at large, doubting of its success, we confidently predicted its triumph. The first week of October has made a great change in affairs. The interior arrangements of this splendid edifice—its museums—its theatres—its costly apparatus for every kind of scientific instruction—and, above all, the specimens of the medical professors already exhibited, have worked a wonderful revolution in the public mind! At each succeeding lecture there was an increase of the ARISTOCRACY—and it may truly be said that—

Those who came to scoff, remain'd to pray.

The number of medical pupils which have already entered to the different classes, is very great indeed—and has tripled the most sanguine expectations of the best friends of the University. The fact is, that no private school of medicine can possibly compete with the advantages and facilities afforded by this University. Clinical instruction will be amply afforded at the MIDDLESEX HOSPITAL and the UNIVERSITY DISPENSARY, both of which are within ten minutes'

walk of the Institution—so that there is not a particle of foundation for the objections which have been propagated on this particular point of the University arrangements. We all know that it is not the mere size of an hospital which constitutes it the best theatre for instruction. If any deduction can be drawn from size of hospital and number of pupils, we would say that the smaller the hospital, and the fewer the pupils, the greater the clinical instruction, provided it be properly taught. At all events, the noscomial establishments above-mentioned, are amply sufficient for the present—and perhaps for ever. As for the cry about religious instruction, it is all hypocrisy. Religious instruction *is* provided for all—and care is taken that those pupils who neglect their Sunday duties to their Creator, shall be reported to their parents. What more can be desired? It is true that no one religious creed is forced upon all—but the Protestant pupil will be expected—indeed in some degree compelled, to attend to the religious instruction of his pastors; while the Dissenter has the option, as he ought to have, of worshipping GOD according to the ideas inculcated by his parents.

But let it never be forgotten, that the main object of such an institution as this, is the concentration under one roof of eminent teachers not only of medical, but of all the other sciences, as well as literature. It is the want of the auxiliary sciences, and the deficiency of general literature, that has been the great drawback on the mass of medical practitioners. It is in vain to aspire at any thing like equality of rank in the profession, till there is an approach at least towards an equality of education. This last being equalized, talent, moral character, and industry, would generally find their level. Till a better education is afforded to medical youth, there will be feuds, dissentions, illiberality, and every kind of degradation going on, to the disgrace of a learned faculty. The following passage from Dr. Conolly's eloquent address is highly entitled to a permanent record here.

"It is hardly necessary for me to observe, that a gentleman practising the higher branches of a liberal profession is expected to have a general acquaintance with modern literature, and some knowledge of what are called the Fine Arts. But it will also prove highly serviceable to him to have studied such parts of Natural Philosophy as explain some of the properties, functions, and capacities of the living body; he will sometimes find it necessary to direct his thoughts to the Philosophy of the Human Mind; his information will be much increased by an acquaintance with the very interesting studies of Comparative Anatomy and Zoology: neither should he be ignorant of Mathematics; and he will often be materially assisted by possessing the accomplishment of Drawing. These acquirements, if they do not all constitute indispensable parts of a complete medical education, may at least precede it with great benefit to the student. The habits of attention which such studies favour, and the store of ideas with which they furnish the student, strengthen, by exercising, his mind; and enable him to enter upon with less difficulty, and to comprehend more readily, the anatomy and physiology of the human body, and whatever relates to the practice of physic and surgery.

"The studies which I have enumerated (for I have omitted many which have sometimes been insisted on) are not at all beyond your reach, provided your early years have been well spent, and you have learned to 'pick up the fragments of your time;' nay, they may be graced and set off with many accomplishments, provided you have no attachment to low and debasing pursuits; provided that your ambition is a well regulated and steady principle, arising from your desire to do what is useful and good; and that your associates and even your amusements are well chosen.

"I need not, I am sure, dwell on the advantage, now first known in London, of an University in which will be presented opportunities for the cultivation of any or of all the parts of knowledge which I have mentioned; situated too, in the midst of an intellectual capital, in which the student can never be driven, by the proscription of elegant and rational amusements, or the want of agreeable and virtuous society, to throw away his early life in low debauchery or vice;—an institution in which the mere parade of learning, or the most laborious perversion of talent, will be far less considered than the attainment of useful knowledge;—an institution in which it is professed, as

I solemnly believe, without reserve or equivocation, that no sect, no party, no persuasion, no difference of rank, or fortune, or opinion, will be a bar to all the academical honours which a pupil may merit, or which can here be bestowed. You must be very inattentive to what is passing around you, if you are not convinced that the careful culture of the mind was never more necessary than it now is, for the preservation of the rank in which you find yourselves, or for the attainment of a higher. Nor will you ever find that your acquisitions are barely equal to the expectations with which your efforts were commenced. Mental industry is always abundantly rewarded. New rays of intelligence, and clearer views of your duty, will be communicated to you from every side; and you will experience, I trust, that the cultivation of true knowledge has not only informed your understanding, but exalted your whole character."

There are some advantages* annexed to this University which, though they may, at first sight, appear to be more of an animal than an intellectual nature, are not so in reality. We allude to the facility afforded to the students of dining together, IN THE UNIVERSITY, and at an extremely small expence (one shilling, we believe) instead of wasting their time, and contaminating their morals in Taverns or Chop-houses. This is a circumstance of the very highest importance. By means of this accommodation, students will be enabled to embrace nearly double the quantity of elementary instruction which they now do, while running from one teacher to another, at various hours of their separate lectures. But this is not all. By dining together, the students become acquainted with each other—the more cultivated and intellectual, naturally and inevitably exert an influence over the others—intelligence is diffused—manners are refined—emulation is fostered—and, in fine, more liberal sentiments are engendered, which prepare the student for mixing with the world, in the first instance, and render his passage through it smoother afterwards.

The question has been widely asked—will the new University *injure* the other schools of medicine in this metropolis? This is a question which we do not feel ourselves called upon to discuss—nor do we think it a question which ought to be entertained. The *consequences* of fair and honorable competition, however disastrous they may prove to individuals, do not deserve the appellation of *injuries*. The great hospitals of London will always attract great crowds of students, not only on account of the number of diseases collected there, but the celebrity of the medical officers attached to those establishments. We hope the day is not far distant when a University education will be combined with, or rather precede attendance on most of the metropolitan hospitals. If the new institution is found to deprive many of the insulated teachers of their pupils, we should deplore the occurrence, in a personal point of view, though we cannot but believe that the public good and the respectability of the profession will be thereby advanced in the end. The stream of medical education is now split into such a number of petty rills, that it is quite ridiculous to peruse the catalogue. It is utterly impossible that such a multiplication of schools can be compatible with success or greatness in any one of them. They must either coalesce and flourish—or divide and dwindle into insignificance.

P. S. We had not the good fortune to be present at the introductory lecture on chemistry by Professor Turner; but we understand it was very favourably received by a most numerous audience.

We may take this opportunity of explaining a mistake or misconception which has gone abroad, respecting the University of Edinburgh, which it is said will refuse the London University certificates, for graduation there. Certainly it will. It never took the time of London lectures as *University time*. But one year out of the four is allowed to *any place of study*, provided the student attends a *public hospital* for six months or more of that year. Thus the lectures at the University of London, and six months attendance at Middlesex Hospital, for example, will count as one year out of

* We are happy to learn that the Council have passed a resolution, that the students at the University, shall not pay more than six guineas for each anatomical subject. The rest of the expense they will bear from the public fund.

the four, for graduation at Edinburgh. *See the 23d volume of the Ed. Med. and Surg. Journal, p. 426, where this point is clearly defined.*

---

### XVI.

### NEW CURRICULUM OF THE APOTHECARIES' COMPANY.

